# Synthesis of the evidence on the impact of pre-operative direct oral anticoagulants on patient health outcomes after hip fracture surgery: rapid systematic review

**DOI:** 10.1007/s00068-022-01937-8

**Published:** 2022-03-11

**Authors:** Rebecca J. Mitchell, Sophie Jakobs, Nicole Halim, Hannah Seymour, Seth Tarrant

**Affiliations:** 1 Australian Institute of Health Innovation, Macquarie University, Level 6, 75 Talavera Road, Sydney, NSW 2109 Australia; 2 Fiona Stanley Hospital, Robin Warren Drive, Murdoch, WA Australia; 3 John Hunter Hospital, New Lambton Heights, NSW Australia; 4 Univeristy of Newcastle, Callaghan, NSW Australia

**Keywords:** Direct oral anticoagulant, Hip fracture, Surgery, Complication, Health outcome

## Abstract

**Purpose:**

To synthesise the evidence on the impact of pre-operative direct oral anticoagulants (DOACs) on health outcomes for patients who sustain a hip fracture.

**Method:**

A rapid systematic review of three databases (MEDLINE, Embase and Scopus) for English-language articles from January 2000 to August 2021 was conducted. Abstracts and full text were screened by two reviewers and articles were critically appraised. Data synthesis was undertaken to summarise health outcomes examined for DOAC users versus a no anticoagulant group. Key information was extracted for study type, country and time frame, population and sample size, type of DOACs, comparator population(s), key definitions, health outcome(s), and summary study findings.

**Results:**

There were 21 articles identified. Of the 18 studies that examined time to surgery, 12 (57.1%) found DOAC users had a longer time to surgery than individuals not using anticoagulants. Five (83.3%) of six studies identified that DOAC users had a lower proportion of surgery conducted within 48 h Four (40.0%) of ten studies reporting hospital length of stay (LOS) identified a higher LOS for DOAC users. Where reported, DOAC users did not have increased mortality, blood loss, transfusion rates, complication rates of stroke, re-operation or readmissions compared to individuals not using anticoagulants.

**Conclusions:**

The effect of DOAC use on hip fracture patient health was mixed, although patients on DOACs had a longer time to surgery. The review highlights the need for consistent measurement of health outcomes in patients with a hip fracture to determine the most appropriate management of patients with a hip fracture taking DOACs.

**Supplementary Information:**

The online version contains supplementary material available at 10.1007/s00068-022-01937-8.

## Introduction

Sustaining a hip fracture is a serious injury for older adults aged ≥ 65 years, as the injury typically requires surgery, can result in ongoing mobility issues, reduced health-related quality of life (HRQoL) or death [1–4]. Much of the evidence indicates that hip fracture surgery should be performed within 1 or 2 days after hospital admission to achieve the best health outcomes, and reduce hospital length of stay (LOS), the likelihood of complications, and mortality [5–8]. However, many older adults with a hip fracture have underlying chronic comorbid conditions, such as thromboembolic disease or atrial fibrillation, that are managed with antithrombotic medication [9]. Traditionally, the use of vitamin K antagonist (VKA) anticoagulants, such as warfarin, has necessitated reversal of their effects pre-operatively to reduce the patient’s international normalized ratio (INR) which may lead to delayed surgical intervention [10, 11].

Unlike VKAs, the use of direct oral anticoagulants (DOACs), including factor Xa inhibitors (i.e., apixaban, rivaroxaban, edoxaban) and a direct thrombin inhibitor (i.e., dabigatran), has the benefit of predictable pharmacokinetics without the need for regular monitoring [10]. However, the use of DOACs has resulted in acknowledged variations in practice in relation to health utilisation outcomes, such as time to surgery, after a hip fracture [10, 12]. While national clinical guidelines recommend hip fracture surgery within 48 hof hospital admission [13–15], there are few consistent guidelines around the pre-fracture use of DOACs and hip fracture surgery.

It is unclear whether the timing of surgical intervention for hip fracture patients prescribed DOACs adversely affects patient or health utilisation outcomes, such as blood loss or hospital LOS, respectively. Any delay to surgery needs to be balanced against potential increased risk of patient complications such as delirium, infection or thromboembolism. The HIP ATTACK study demonstrated that operating within 6 h of presentation with a hip fracture had no detrimental effect and was associated with a lower risk of delirium, urinary tract infection, and moderate to severe pain scores on days 4–7 compared to standard care [16]. Early surgery in HIP ATTACK led to faster mobilisation, a shorter hospital LOS and no difference in mortality compared to usual care [16].

Surgical delays to allow medical optimisation of patients taking pre-fracture anticoagulation treatment aim to reduce intra- and post-operative blood loss [17] and may be necessary to deliver safe regional anaesthesia [18]. As the use of DOACs is increasing among older adults [19], and with the number of hip fractures worldwide estimated to rise to 6.26 million by 2050 [20], whether there is evidence of a detrimental effect on health outcomes of older adults taking DOACs pre-operatively needs to be collated and synthesised. The aim of this systematic rapid review is to synthesise the current evidence on the impact of pre-operative DOACs on patient health outcomes for patients who sustain a hip fracture. This will inform future research, audit and guideline development.

## Methods

This rapid systematic review synthesises the evidence on the impact of pre-operative DOACs on the health outcomes of older adults who underwent hip fracture surgery. The review records the type of DOACs examined, whether any comparator population(s) were included, the type of primary and secondary health outcomes examined, and summarises the findings of each study.

### Definitions

Research articles were included in the rapid review if they examined health outcomes of patients who underwent hip fracture surgery. Articles were included if the population in the studies was primarily aged ≥ 60 years, was admitted to hospital after a hip fracture (e.g. intracapsular, trochanteric or subtrochanteric), and underwent surgery for the hip fracture (e.g. intramedullary nail or hip screw, hemiarthroplasty, total hip replacement).

The research articles included had to evaluate the impact of pre-operative DOACs (i.e., rivaroxaban, apixaban, dabigatran, edoxaban) on patient health outcomes. Comparator populations could include patients not taking DOACs, patients taking VKAs (e.g., warfarin) or patients taking oral platelet inhibitors (PAIs) (e.g., aspirin, clopidogrel, ticagrelor).

Patient health outcomes included those relating to health-care utilisation, such as time to surgery, mortality, hospital LOS, intensive care unit (ICU) LOS, blood loss, need for a blood transfusion, or post-operative complications (e.g., infection, bleeding, pulmonary embolism, deep vein thrombosis). For the purposes of this rapid review, measures of health outcomes related to life post-discharge, such as mental health, HRQoL, or ability to perform activities of daily living (ADLs), were excluded.

### Data sources and eligibility criteria

A systematic search was conducted using three databases: MEDLINE, Embase, and Scopus. The search strategy was developed with a university librarian and included the following search terms: (anticoagulant* OR ‘oral anticoagulant’ OR ‘DOAC*’ OR Rivaroxaban OR Apixaban OR Dabigatran OR Edoxaban) AND ABS (‘hip fracture’ OR ‘hip surgery’) AND (postoperative* OR mortality OR ‘time to surgery’ OR ‘length of stay’ OR ‘complication*’ OR bleed* OR ‘blood loss’ OR embolism OR thrombosis OR ‘intensive care*’) (see Appendix 1 for full search strategy).

Articles were excluded if patients did not have surgery after their hip fracture, patients were not taking DOACs pre-operatively (other than comparator populations), if the article was a systematic review, other type of review, a single case report, a study protocol, or if there was insufficient detail regarding the health outcome(s) examined. Results were limited to English-language articles that were published in peer-review journals from 1 January 2000 to 31 August 2021. Snowballing of article reference lists and review of co-author repositories was conducted to identify any potential articles not previously identified.

### Abstract screening

The full citation information including title and abstract of each article identified during the database searches was imported into Endnote X20 and duplicates removed. The abstracts were independently assessed for inclusion by two reviewers (SJ, NH), who met regularly to discuss any uncertainties. If the abstract did not report that the research evaluated the impact of pre-operative DOACs on patient health outcomes after hip fracture surgery it was excluded. Both reviewers (SJ, NH) screened 15.0% of the articles and the interrater percent agreement was 82.9%. Any disagreements on abstract inclusion were discussed with a third reviewer (RM) and consensus achieved.

### Full-text screening, data extraction and quality review

The full text of each article was assessed by two reviewers (SJ, NH), if the article was included in the abstract review stage. Any article that did not meet the inclusion criteria was excluded. For articles that met the inclusion criteria, key information was extracted from each article during the full-text review by two reviewers (SJ, NH), including: authors and publication year; review objective/aim; study type, country and time frame examined, population and sample size, type of DOACs examined, comparator population(s), health outcome(s), and summary study findings. Data extraction results were independently appraised for accuracy by a third author (RM). The methodological quality of the articles was assessed by two reviewers (SJ, NH) and appraised by a third reviewer (RM) using the Critical Appraisal Skills Programme (CASP) cohort [21] or case–control [22] study checklists, where applicable. The quality of retrospective matched case-comparison studies was assessed with the case–control study checklist. Any clarifications regarding methodological quality were discussed between reviewers.

### Data synthesis

The information on the included studies in the data extraction table was compared and a data synthesis was undertaken by one reviewer (RM) and appraised by two reviewers (HS and ST). The data synthesis involved identifying the most common health outcomes examined for DOAC users versus the no anticoagulant use comparator group. The findings for each health outcome were summarised as to whether DOAC users had a worse outcome than a no anticoagulant comparator population, where possible. The data extraction and data synthesis results were examined by two authors (HS and ST) and whether any recommendations could be made regarding the pre-operative use of DOACs and the timing of hip fracture surgery based on the existing research evidence was considered (Supplementary Table 1).

## Results

**Fig. 1:**
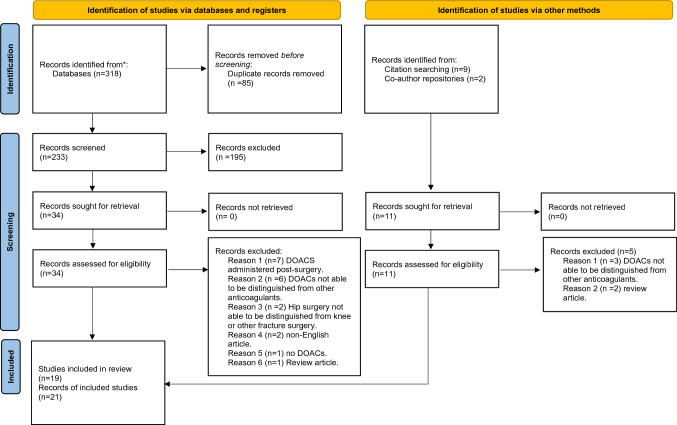
PRISMA flow diagram

A total of 318 articles were identified during the database searches. After removing duplicates, 233 articles remained. After abstract review, 34 full-text articles were examined, along with 11 articles from snowballing. A final 21 articles were included in the rapid review (Fig. 1).

### Study type, country and comparator population

Around three-quarters (76.2%) were retrospective cohort studies, with five (23.8%) case–control/comparison designs. The number of patients taking DOACs in the studies ranged from 11 to 1063, with a median of 33 patients. Three (14.3%) studies each were conducted in Australia, the United Kingdom (UK), Israel, and the United States (US), two (9.5%) each in Canada, Germany and Norway, and one (4.8%) study each in Austria, Denmark and Italy. Ten (47.6%) studies used two comparison groups, seven (33.3%) used one comparison group, two (9.5%) studies used three comparison groups and two (9.5%) studies used four comparison groups. The comparison groups involved patients not taking any anticoagulants pre-surgery (*n* = 18 groups), patients taking VKAs (*n* = 14 groups), and patients taking PAIs (*n* = 7 groups).

### Patient age and common health outcomes

The age inclusion criteria varied for patients, eight (38.1%) studies included patients aged ≥ 65 years, four (19.0%) studies included patients aged ≥ 60 years, one (4.8%) study included patients aged > 70 years, five (23.8%) studies did not specify their patient age inclusion criteria, but the mean age of patients in these studies was in the mid-80 s. Where indicated, the mean patient age ranged from 80.7 to 85.0 years and median age ranged from 83.5 to 86.0 years (Table 1).

**Table 1:** Characteristics of studies examining the impact of DOACs on patient health outcomes after hip fracture surgery

Authors and publication year	Objective/aim	Study type	Country/study time frame	DOAC (s) examined (number of patients by drug)	Comparison population(s)	Population and sample size
Bruckbauer et al. 2019 [48]	To assess the impact of DOAC intake compared with coumadin (COU) in hip fracture (HF) patients	Retrospective cohort	Salzburg, Austria Jan 2015–May 2017	Any DOAC (*n* = 13 dabigatran, *n* = 34 rivaroxaban, *n* = 7 apixaban)	1. COU 2. no antithrombotic therapy (no-ATT)	Patients ≥ 65 years with a median age of 83.5 years [range 76–89] *n* = 54 DOAC; *n* = 59 COU; *n* = 207 no-ATT
Cafaro et al. 2019 [49]	To establish TTS among non-anticoagulated and anticoagulated patients taking either vitamin K antagonist (VKA) or DOACs	Retrospective cohort	Canada, 1 July 2016–31 Dec 2017	Any DOAC (*n* = 6 dabigatran, *n* = 21 apixaban, *n* = 4 rivaroxaban)	1. VKA 2. no anticoagulant	Patient inclusion was age ≥ 18 years, but mean age was 84 years [range 29–98] *n* = 31 DOAC; *n* = 28 VKA; *n* = 413 no anticoagulant
Creeper et al. 2020 [50]	To investigate the effect of DOAC therapy on time to surgery and patient outcomes, and to explore the impact of different pre-operative protocols on surgical delay	Retrospective cohort	Australia, 1 Jan 2017–31 Dec 2017	Any DOAC (*n* = 8 dabigatran, *n* = 44 apixaban, *n* = 29 rivaroxaban, *n* = 1 edoxaban)	1. Warfarin 2. antiplatelets 3. dual anticoagulation and antiplatelet 4. no anticoagulation or antiplatelet	Patient inclusion age was not specific, but median age was 84 years [interquartile range: 76–89] *n* = 82 DOACs; *n* = 63 warfarin; *n* = 366 antiplatelets; *n* = 13 dual anticoagulation and antiplatelet; *n* = 714 no anticoagulation or antiplatelets
Daugaard et al. 2019 [47]	To examine if pre-operative antithrombotic treatment was associated with increased use of blood transfusion and 30-day mortality following hip fracture surgery	Retrospective cohort	Denmark, 1 Jan 2005–31 Dec 2016	Any DOAC—both current (i.e., at least one prescription ≤ 90 days prior to surgery) and former (i.e., redemption of one prescription 91–365 days prior to surgery) users (unspecified number by DOAC type)	1. VKA; 2. antiplatelets including acetylsalicylic acid; 3. non-user of DOACs in the year prior to surgery	Patients ≥ 65 years. Mean age not specified *n* = 1063 DOAC, *n* = 4162 VKA, *n* = 24,567 antiplatelets; *n* = 73,507 non-DOACs
Franklin et al. 2018 [37]	To evaluate the pre-hospital use of DOACs on the outcomes of early surgical fixation of geriatric HF	Retrospective case–control	US, 2010–2015	Any DOAC (*n* = 6 rivaroxaban, *n* = 5 apixaban *n* = 8 dabigatran)	1. Controls (unspecified criteria, but excluded patients on plavix or coumadin or > 81 mg of daily aspirin) matched on age ± 3 years, sex, and operation type (i.e., hemiarthroplasty, cephalomedullary nail (CMN), sliding hip screw	Patients aged 60–89 years who underwent HF surgery within 48 h of admission. Mean age not specified *n* = 19 DOACs *n* = 76; controls
Frenkel Rutenberg et al. 2018 [51]	To assess outcomes of patients treated with VKAs or DOACs undergoing surgical treatment for fragility HF	Retrospective cohort	US, Jan 2012–Jun 2016	Any DOAC (*n* = 18 dabigatran, *n* = 16 rivaroxaban, *n* = 13, apixaban)	1. VKAs 2. no-anticoagulation	Patients > 65 years with a mean age ~ 82 years *n* = 47 DOACs; *n* = 103 VKAs; *n* = 646 no anticoagulation
Gosch et al. 2020 [52]	To compare the short-term outcome of older hip fracture patients without oral anticoagulation, VKAs and DOACs	Retrospective case–control	Germany, Feb 2017–Jun 2018	Any DOAC (unspecified DOAC type)	1. No anticoagulants 2. VKA	Patients > 70 years Patient mean age was 83.9 years *n* = 26 DOACs, *n* = 15 VKA; *n* = 61 no anticoagulants
Hourston et al. 2020 [42]	To assess whether HF patients admitted on Warfarin or DOACs were at risk of operative delay, prolonged LOS, or increased mortality	Retrospective cohort	UK, Oct 2014–Dec 2016	Any DOAC (*n* = 19 rivaroxaban, *n* = 8 apixaban, *n* = 5, dabigatran)	1. No anticoagulants 2. Warfarin	Inclusion age unspecified Patient mean age was 85 years *n* = 32 DOAC, *n* = 83 warfarin, *n* = 729 no anticoagulants
King et al. 2020 [38]	To investigate the effect of DOACs on patient outcomes receiving early (< 48 h) versus delayed (> 48 h) HF surgery	Retrospective cohort	Queensland, Australia; Jan 2012–Dec 2017	Any DOAC (*n* = 5 apixaban, *n* = 8 dabigatran, *n* = 15 rivaroxaban)	1.No DOACs matched to TTS < 48 h on age, ASA grade, sex, surgery type (i.e., arthroplasty vs. other fixation), time to surgery (< 48 h) and dementia status	Inclusion age unspecified Mean age ~ 84 years *n* = 28 DOAC (*n* = 17 early surgery and *n* = 11 late surgery), *n* = 56 no DOACs
Leer Salvesen et al. 2020 [43]	To determine whether DOAC users have delayed HF surgery compared to non-DOAC users and whether hospital LOS, mortality, re-operations and bleeding complications were influenced by use of DOACs	Retrospective cohort	Norway, Dec 2016–Dec 2017	Any DOAC (unspecified number by DOAC type)	1. No anticoagulants	Patients aged > 60 years, with a mean age of 82.1 years *n* = 47 DOACs; *n* = 267 no anticoagulants
Lott et al. 2019 [12]	To evaluate whether patients with HFs receiving platelet aggregation inhibitors (PAI) and DOACs treated within 48 h of admission had worse surgical and clinical outcomes than those whose surgery was delayed > 48 h	Retrospective cohort	US, Oct 2014–Sep 2016	Any DOAC (*n* = 4 dabigatran, *n* = 10 rivaroxaban, *n* = 15 apixaban)	1. PAI including clopidogrel and aspirin	Patients ≥ 55 years. However, mean age was ~ 83 years *n* = 29 DOACs; *n* = 49 PAI (*n* = 38 clopidogrel; *n* = 11 aspirin)
Mahmood et al. 2021 [40]	Review whether taking PAIs or anticoagulants was associated with increased mortality for HF patients; and to evaluate the mortality and complication rates for patients taking these agents who underwent early (< 24 h) surgery	Retrospective cohort	UK, Jan 2016–Jan 2019	Any DOAC (unspecified number by DOAC type)	1. Control group (no PAI or anticoagulants 2. PAI (i.e., aspirin or clopidogrel) 3. Warfarin	Patients aged ≥ 60 years with a mean age of 82.1 years *n* = 69 DOACs; *n* = 617 controls; *n* = 260 PAI; *n* = 92 warfarin
Mullins et al. 2018 [26]	To determine whether not waiting for the elimination of DOACS has an effect on the amount of peri-operative bleeding in HF patients	Retrospective case–control	UK, Jan 2015–March 2017	Any DOAC (*n* = 14 apixaban, *n* = 5 dabigatran, *n* = 44 rivaroxaban)	1. Patients not taking DOACs or warfarin (matched on age ± 5 years, sex, operation, American Society of Anesthesiologists (ASA) grade	Patients aged ≥ 60 years with a mean age of 85 years *n* = 63 DOACs; *n* = 62 no DOACs
Rostagno et al. 2021 [41]	To investigate the effects of ongoing treatment with DOACs on TTS and on in-hospital clinical outcomes in patients with HF	Retrospective case–control	Italy, Jan 2016–Jan 2019	Any DOAC (*n* = 28 dabigatran, *n* = 19 rivaroxaban, *n* = 24 apixaban, *n* = 3 edoxaban)	1. Patients not taking anticoagulants (matched on age, sex, fracture type, ASA grade	Elderly patients, age unspecified. Mean age was ~ 84 years *n* = 74 DOACs; *n* = 206 no-anticoagulants
Saliba et al. 2020 [44]	Assess the association between pre-operative DOACs use and adverse outcomes in elderly patients with HF	Retrospective cohort	Israel, 1 Jan 2014–31 Dec 2018	Any DOACs (*n* = 129 apixaban, *n* = 71 rivaroxaban, *n* = 47 dabigatran)	1. VKAs 2. no anticoagulants	Patients ≥ 65 years with a mean age of 82.2 years *n* = 247 DOACs; *n* = 163 VKAs; *n* = 3,008 no anticoagulants
Scherman et al. 2019 [45]	To compare estimates of peri-operative blood loss and mortality between HF patients taking DOACs and no anticoagulation	Retrospective cohort	Israel, 2011–2016	Any DOAC (apixaban, rivaroxaban and dabigatran, unspecified number by DOAC type) Could have concurrent use of aspirin and PAI (clopidogrel, prasugrel, ticagrelor)	1. control with no anticoagulant use 2. Coumadin	Patients ≥ 65 years who had closed reduction internal fixation (CRIF) or hemiarthroplasty (HA) with a mean age ~ 82 years *n* = 89 DOACs; *n* = 1,466 no anticoagulation; *n* = 159 coumadin (*n* = 18 DOACs taking aspirin and *n* = 5 taking clopidogrel)
Schuetze et al. 2019 [46]	To determine the effect of DOACs on HF patients which received a proximal femur nail anti-rotation (PFNA) within 24 h after trauma	Retrospective cohort	Germany, Jan 2013– Dec 2017	Any DOACs (unspecified number by DOAC type)	(1) no anticoagulation; (2) acetylsalicylic acid (ASS); (3) PAI; (4) VKA	Patients of all ages, but mean age was 80.7 years *n* = 52 DOACs; *n* = 146 no anticoagulation; *n* = 74 ASS; *n* = 30 PAI; *n* = 25 VKA
Shani et al. 2021 [53]	To investigate if patients treated with DOACs have delayed HF surgical compared to patients on no anticoagulants or Warfarin, and if there is an impact on TTS, LOS and mortality	Retrospective cohort	Israel, 1 Jan 2014–31 Dec 2017	Any DOAC (unspecified number by DOAC type)	1. No oral anticoagulant; 2. warfarin	Patients > 65 years with a mean age of ~ 83 years *n* = 415 DOACs; *n* = 5,102 no anticoagulants; and *n* = 311 warfarin
Tarrant et al. 2020 [39]	To investigate how DOACs affect surgical timing and peri-operative outcomes	Retrospective case–control	Australia; 2011–2018	Any DOACs (*n* = 56 apixaban, *n* = 18 dabigatran, *n* = 38 rivaroxaban)	1. control group not taking antithrombotic medication and matched on age, sex, and year of admission	Patients aged ≥ 65 years, with a mean age of 84.3 years *n* = 112 DOACs; *n* = 112 controls
Tran et al. 2015 [54]	To determine how anticoagulation with VKA or DOAC affects TTS	Case–control	Canada, 1 Jan 2010–24 March 2014	Any DOAC (*n* = 22 dabigatran, *n* = 4 rivaroxaban, *n* = 1 apixaban)	1. VKAs 2. no anticoagulants matched on age and sex	Age inclusion criteria not specified, but median age 86 years *n* = 27 DOACs; *n* = 260 no anticoagulants; *n* = 233 VKAs
Viktil et al. 2019 [55]	To determine serum concentrations and elimination rates of DOACs in HF patients and TTS	Prospective cohort pilot study	Oslo, Norway (6-month period, dates unspecified)	Any DOAC (*n* = 2 dabigatran, *n* = 3 rivaroxaban, *n* = 6 apixaban)	1. Warfarin 2. PAI	Patients ≥ 65 years, with a median age 84 years (*n* = 11 DOACs; *n* = 14 warfarin; *n* = 50 PAI)

Authors and publication year	Blood loss definition	Time to surgery definition	Type of anaesthesia	Hours prior to surgery DOACs ceased	Use of reversal agents	Health outcome(s) examined (primary and secondary outcomes)	Study findings (primary and secondary)
Bruckbauer et al. 2019 [48]	Drainages from intensive care unit (ICU) admission to discharge	Time from admission to surgery	NR	NR	Specified in secondary study findings	Primary: (1) transfusion requirements; (2) post-operative bleeding rate Secondary: (1) time to surgery (TTS); (2) use of reversal agents; (3) intensive care unit (ICU) length of stay (LOS); (4) mortality	Primary: (1 and 2) no difference in blood less through drainages for DOAC or COU Secondary: (1) TTS longer for DOAC group: 37% on DOACs, 51% on COU, and 55% no-ATT had surgery within 24 h (2) Reversal agents: 78% on COU received vitamin K and 4 patients received PCC One patient on DOAC received prothrombin complex concentrate (PCC) and one patient on dabigatran received idarucizumab (3) No differences in ICU LOS (4) No differences for in-hospital mortality
Cafaro et al. 2019 [49]	International Society on Thrombosis and Haemostasis (ISTH) Any major bleeding from presentation to discharge including surgical bleeding Pre-operative major bleeding limited to the pre-operative period	Time from hospital admission to surgery	NR	NR	Peri-operative use of PCC, vitamin K, idarucizumab and/or plasma, unspecified further	Primary: (1) TTS Secondary: (1) surgical delay; (2) hospital LOS; (3) acute venous thromboembolism (VTE), pulmonary embolism PE); (4) any major bleeding and pre-operative major bleeding; (5) stroke; (6) in-hospital mortality	Primary: (1) Median TTS was longer in VKA (64 h) and DOAC (61 h) vs. no anticoagulant (44 h). No difference in TTS for VKA (64 h) vs. DOAC (61 h) Secondary: (1) No VKA and 39% of DOACs had surgery within 48 h vs. 60% no anticoagulant (2) Similar LOS for VKA, DOAC and no anticoagulant; 15.6, 16.1, 16.6 days, respectively (3) No difference in VTE or PE for no anticoagulant, VKA or DOAC (4) No difference in any major bleeding for DOAC (80.0%) vs. VKA (75.0%) vs. no anticoagulant (84.9%) Pre-operative major bleeding higher in DOAC (24.0%) vs. VKA (14.2%) vs. no anticoagulant (10.0%) (5) No difference in stroke between groups (6) No difference in in-hospital mortality for DOAC (6.5%), VKA (7.1%) and no anticoagulant (3.4%)
Creeper et al. 2020 [50]	Change in haemoglobin (Hb) (g/L) defined as the difference between the maximum recorded Hb level up to 48 h pre-operatively to the minimum level recorded within 72 h post-operatively	Time of first presentation to any hospital (regardless of location and ability to perform correctional surgery) to the time of operation	NR	NR	NR	Primary: (1) TTS Secondary: (1) post-operative change in haemoglobin; (2) transfused packed red blood cell units 2 days pre-operatively to 3 days post-operatively; (3) 30-day mortality	Primary: (1) median TTS was longer for DOAC (43.9 h) vs. warfarin (27.9 h) and TTS for DOACs vs. other comparator populations was not conducted Secondary: (1) there was no difference in change in haemoglobin between DOAC vs. warfarin and change in haemoglobin for DOACs vs. other comparator populations was not conducted (2) There was no difference in transfusion requirements between DOAC vs. warfarin and transfusion requirements for DOACs vs. other comparator populations was not conducted (3) There was no difference in 30-day mortality for DOAC vs. warfarin and comparison of 30-day mortality for DOACs vs. other comparator populations was not conducted
Daugaard et al. 2019 [47]	NR	Time from hip fracture admission to surgical procedure	NR	NR	NR	Primary: (1) red blood cell transfusion within 7 days (2) 30-day mortality Secondary: (1) transfusion and TTS; (2) 30-day mortality and TTS	Primary: (1) compared to non-DOAC users, current DOAC users had a 7% higher adjusted relative risk of a blood transfusion and former DOAC users had no higher transfusion risk (2) Compared to non-DOAC users, both current and former DOAC users had no higher risk of mortality Secondary: (1) compared to non-DOAC users, current DOAC users who had surgery < 24 h had a 14% higher risk of a blood transfusion, and there was no difference for current DOAC users who had surgery between 24 and 36 h or > 36 h. There was no difference for former DOAC users that had surgery < 24 h, between 24-36 h or > 36 h compared to non-DOAC users (2) Compared to non-DOAC users, current and former DOAC users who had surgery < 24 h, between 24 and 36 h or > 36 h. Did not have a higher 30-day mortality compared to non-DOAC users
Franklin et al. 2018 [37]	NR	NR	One DOAC and three controls received spinal anaesthesia as an adjunct to general endotracheal anaesthesia. One control received spinal anaesthesia with MAC sedation. Otherwise all had general endotracheal anaesthesia without adjuncts	Mean (SD) estimated time between most recent ingestion and surgery = 39.5 (14.7) hours	NR	Peri-operative outcomes: (1) estimated blood loss (EBL) (mL) (2) transfusion rate (3) TTS (h) Post-operative outcomes: (4) LOS (5) Peri-operative complications, including haematoma formation, persistent serous drainage, thromboembolic events, or need for re-operation (6) Readmission rate (7) in-patient, 30-day, 90-day, and 1-year survival	Peri-operative outcomes: (1 and 2) no difference for EBL, transfusion rates, or blood volume transfused (3) DOACs (28.9 h) within 48 h of admission had longer TTS than controls (21.4 h) Post-operative outcomes: (4, 5) No difference in LOS or peri-operative complication rates (6) DOACs (21%) readmitted at higher rate vs. controls (5.3%) (8) No difference in survival at in-patient, 30 days, 90 days, or at 1 year. Survival at 1 year was DOAC (70.6%) vs. controls (59.1%)
Frenkel Rutenberg et al. 2018 [51]	Need for blood transfusion during hospitalisation	Time from admission to surgery	NR	NR	NR	Primary: (1) in-hospital and 1-year mortality Secondary: (1) TTS within 48 h; (2) complication (e.g. infection, cardiovascular, pulmonary, renal, neurological, thromboembolic); (3) blood transfusions; (4) readmission within 1 year	Primary: (1) No difference for in-hospital or 1-year mortality Secondary: (1) Lower proportion of DOAC (51%) and VKAs (59%) had TTS within 48 h vs. no-anticoagulation (92%) (2) No difference in-hospital complications (3) No difference in blood transfusions (4) No difference in readmissions
Gosch et al. 2020 [52]	Major bleeding defined as decrease in haemoglobin level of 2 g per decilitre or more over a 24 h period, transfusion of ≥ 2 units of packed red cells, bleeding at a critical site (intracranial, intraspinal, intraocular, pericardial, intraarticular, intramuscular with compartment syndrome or retroperitoneal) or fatal bleeding	NR	NR	NR	NR	Primary: (1) in-hospital mortality; (2) TTS; (3) LOS; (4) ICU and ICU LOS; (5) place of discharge; (6) mobility; (7) complications (incl revision surgery, wound infections, urinary tract infection, pneumonia, myocardial infarction, stroke, thromboembolic events, falls during hospital stay, minor and major bleeding, delirium); (8) blood loss; (9) need for packed red cells, thrombocytes, prothrombin complex concentrate, fresh frozen plasma	Primary: (1) no difference in in-hospital mortality for DOAC (3.8%) vs. VKA (20%) or no anticoagulants (9.8%) (2) TTS longer for DOAC (42.7 h) vs. no anticoagulants (30 h), but no difference to VKA (40.5 h); (3) LOS longer for DOAC (17.2 days) vs. no anticoagulants (12.6 days) but no difference to VKA (14.4 days); (4) no difference in ICU and ICU LOS between DOACs vs. VKA or no anticoagulants; (5) no difference in discharge location; (6) No difference in mobility at discharge; (7) no difference in any complications, except for minor bleeding, which was higher for VKA (33.3%) vs. no anticoagulants (6.6%), but no difference to DOAC (19.2%); (8) no difference in decrease of haemoglobin; (9) no difference in need for packed red cells
Hourston et al. 2020 [42]	NR	Time from admission to theatre	NR	NR	None used	Primary: (1) TTS; (2) LOS; (3) 30-day; 6- and 12-month mortality	Primary: (1) TTS longer for DOAC (29 h) vs. warfarin (27 h) and no anticoagulants (22 h). DOAC not associated with increase in TTS > 48 h Subgroup analysis found apixaban and dabigatran associated with delay > 36 h, but not rivaroxaban. None were associated with a delay > 48 h (2) No difference for LOS (3) Warfarin (22%) lower 30-day survival, but not DOACs (6%) or no anticoagulation (4%). No difference for 6- or 12-month mortality
King et al. 2020 [38]	Measured as greatest haemoglobin value minus lowest haemoglobin value from admission to post-operative day 2	Time from hospital admission to surgery	NR	NR	NR	Primary: (1) Blood transfusion rates; (2) peri-operative blood loss; (3) acute and total LOS; (4) TTS; (5) 30- and 90-day mortality; (6) re-operation; (7) haematoma rates	Primary: (1) no difference in blood transfusion rates (2) No difference in peri-operative blood loss (3) No difference in acute or total LOS (4) TTS longer for early DOAC (32.2 h) than no DOAC (26.0 h) (5) No difference in 30-day mortality. However, late DOAC (36.4%) had higher 90-day mortality vs. early DOAC (0%) (6) No difference in re-operation (7) Nil haematomas
Leer Salvesen et al. 2020 [43]	Intra-operative blood loss estimated by the surgical team Blood transfusions for patients with Hb < 9 g/dL and collected from medical records	Time from admission to surgery	General anaesthesia (*n* = 22 DOACs; *n* = 10 no anticoagulants). Spinal anaesthesia (*n* = 25 DOACs; unspecified for anticoagulants)	NR	NR	Primary: (1) TTS; (2) LOS Secondary: (1) in-hospital, 30-day and 6-month mortality; (2) readmission within 30 days and 6 months; (3) Blood loss during surgery; (4) blood transfusions; (5) wound ooze	Primary: (1) No difference TTS DOAC (28.9 h) vs. no anticoagulants (26.1 h) (2) No difference in LOS DOAC (6.6 days) vs. no anticoagulants (6.1 days) Secondary: (1) No difference in in-hospital (4.3% vs. 3.4%), 30-day (10.6% vs. 12.7%) or 6-month (23.4% vs. 22.1%) mortality for DOAC vs. no anticoagulants, respectively (2) No difference in 30-day or 6-month readmission (3) No difference in mean blood loss during surgery (4) No difference in transfusion rates (5) Wound ooze higher for DOACs (26%) vs. no anticoagulant (5.6%)
Lott et al. 2019 [12]	Surgical blood loss	Early: surgery within 48 h of presentation; or delayed: surgery > 48 h of presentation	General anaesthesia; spinal anaesthesia, unspecified by DOAC or PAI group	NR	NR	Primary: (1) length of surgery; (2) blood loss during surgery (mL); (3) transfusion requirement; (4) complications (i.e., sepsis, pneumonia, DVT/PE, acute myocardial infarction, acute kidney injury, stroke, surgical site haematoma, decubitus ulcer, urinary tract infection, acute respiratory failure, acute anaemia, cardiac arrest, and inpatient mortality); (5) transfer to ICU/ step-down unit	Primary: (1) no difference for surgery length for DOACs or PAIs; (2) no difference for blood loss for DOACs or PAIs; (3) no difference for transfusions for DOACs or PAIs; (4) no difference for DOACs or PAIs for complications for those treated within 48 h or > 48 h of admission; (5) no difference for ICU transfers for patients treated within 48 h or > 48 h (unspecified ICU transfers by DOACs or PAIs)
Mahmood et al. 2021 [40]	Hb levels on admission and the first post-operative day to calculate post-operative Hb drop	Early (i.e., within 24 h of admission) or late (> 24 h of admission)	NR	Aspirin, clopidogrel, DOACs were stopped on admission and surgery was advocated within 36 h, unless patient needed further optimisation or medical workup	Warfarin stopped on admission and 10 mg of intravenous vitamin K given; and INR checked at 6 h. If level > 1.5 further intravenous vitamin K given	Primary: (1) TTS; (2) mortality at 30-days and 1 year; (3) post-operative Hb drop; (4) transfusion rate; (5) wound ooze; (6) infection rate (i.e., prescribed antibiotics); (7) re-operation rate	Primary: (1) no difference in mean TTS (hrs): control (23.5); PAI (24.4); warfarin (29.6); DOAC (28.1) (2) Difference in 30-day mortality: control (4.8%); PAI (12.6%); Warfarin (7.0%); DOAC (9.5%) and 1-year mortality: control (22.4%); PAI (32.3%); warfarin (29.3%); DOAC (29.0%) (3) No difference in post-operative Hb. However sub-group analysis showed difference for DOAC group for TTS < 24 h (20.1 g/L) vs. TTS > 24 h (14.7 g/L) (4) Difference in transfusion rate: control (21.6%); PAI (32.3%); Warfarin (21.7%); DOAC (23.2%) (5) Difference in wound ooze: control (22.2%); PAI (26.9%); warfarin (40.2%); DOAC (24.6%). Sub-group analysis showed difference for PAI group for TTS < 24 h (30.4%) vs. TTS > 24 h (18.4%) and Warfarin group for TTS < 24 h (27.5%) vs. TTS > 24 h (50.0%) (6) No difference in infection rates: control (1.0%); PAI (0.8%); warfarin (0%); DOAC (2.9%) (7) No difference in re-operation rates: control (0.6%); PAI (0.8%); warfarin (0%); DOAC (2.9%)
Mullins et al. 2018 [26]	Peri-operative blood loss and blood transfusions	NR	NR	NR	NR	Primary: (1) TTS; (2) peri-operative change in in Hb concentration; (3) blood transfusion; (4) re-operation; (5) 30-day mortality	Primary: (1) no difference in TTS: DOACs (19 h) v no-DOACs (19 h); (2) no difference in peri-operative change in Hb concentration: DOACs (23 g/L) vs. no-DOACs (23 g/L); (3) no difference in blood transfusion: DOACs (18%) vs. no-DOACs (10%); (4) no difference in re-operation: DOACs (5%) vs. no-DOACs (*n* = 0%) (5) No difference in 30-day mortality: DOACs (2%) vs. no-DOACs (8%)
Rostagno et al. 2021 [41]	Need for blood transfusion	NR	NR	NR	High-risk elective surgery was allowed 48 h after last administration for apixaban, edoxaban and rivaroxaban. Dabigatran timing was related to renal function (creatinine clearance)	Primary: (1) TTS; (2) % treated within 48 h; (3) LOS; (4) % with blood transfusion; (5) in-hospital mortality; (6) intra-operative complication (type not specified); (7) post-operative complications (anaemia, respiratory failure, acute renal failure, pneumonia, sepsis, wound infection, PE, DVT, acute myocardial infarction, acute heart failure, delirium, stroke)	Primary: (1) TTS longer for DOACs (3.6 h) vs. no anticoagulants (2.2 h) (2) Less DOACs (47%) surgery < 48 h vs. no anticoagulants (80%) (3) No difference in LOS: DOAC (14 days) vs. no anticoagulants (14.6 days) (4) No difference for blood transfusion: DOAC (46%) vs. no anticoagulants (41%) (5) No difference in mortality: DOACs (1.5%) vs. no anticoagulants (3.4%) (6) No difference in intra-operative complications (7) No difference in post-operative complications, except for anaemia (Hb < 8.0 g/dl): DOACs (37%) vs. no anticoagulants (12%)
Saliba et al. 2020 [44]	Intra-operative bleeding subjectively estimated by the surgeon	NR	Of the 1108 patients with TTS, 69.9% received general anaesthesia and 30.1% received regional anaesthesia	NR	NR	Primary: (1) all-cause in-hospital mortality and mortality at 30-day, 90-day and 1-year Secondary: (1) LOS; (2) TTS within 48 h; (3) blood transfusion; (4) intra-operative bleeding	Primary: (1) compared to no anticoagulant use, DOACs had lower odds of mortality at 30 and 90 days and at 1 year Secondary: (1) Compared to no anticoagulant use, DOACs and VKAs users had longer LOS (2) Compared to no anticoagulant use, DOAC and VKA users had lower odds of surgery within 48 h (3) Compared to no anticoagulant use, no difference in proportion of DOAC or VKA users who received a blood transfusion (4) Compared to no anticoagulant use, no difference in intra-operative moderate-severe bleeding for DOAC or VKA users
Scherman et al. 2019 [45]	Peri-operative haemoglobin change as the difference between pre- and post-operative Hb levels divided by pre-operative Hb level multiplied by 100 Blood transfusions within 1 week from surgery	Time from admission to surgery	NR	NR	If patient had normal kidney function, patient taking rivaroxaban and apixaban were operated on after 24-36 h. Patients taking dabigatran were operated on 12-24 h after last tablet	Primary: (1) %Hb change; (2) blood transfusions; (3) 30-day and 1-year mortality Secondary: (1) TTS; (2) % of HF surgery within 48 h	Primary: (1) No difference in %Hb change between DOACs and no-anticoagulants: CRIF DOACs (22.6%) and no-anticoagulants (24.0%) and HA DOACs (21.7%) and no anticoagulants (21.0%) (2) No difference for CRIF or HA for blood transfusion rates for DOACs (8.3% & 10.3%, respectively) and no anticoagulants (7.9% & 7.4%, respectively) (3) No difference for CRIF or HA for blood 30-day mortality for DOACs (6.7% & 6.9%, respectively) and no anticoagulants (4.4% & 6.1%, respectively) 1-year mortality was higher for CRIF for DOACs (26.7%) compared to no-anticoagulants (16.1%), but not for HA for DOACs (13.8%) compared to no anticoagulants (21.1%) Secondary: (1) TTS longer for CRIF for DOACs (40.2 h ± 26.9) compared to no anticoagulants (31.2 h ± 22.2) and no difference for HA (DOACs 42.3 h ± 27.3 and no-anticoagulants 36.6 h ± 25.8) (2) No difference in % of HF surgery within 48 h between DOACs and no anticoagulants: CRIF DOACs (74%) and no anticoagulants (82%) and HA DOACs (74%) and no anticoagulants (78%)
Schuetze et al. 2019 [46]	Decision for blood transfusion based on Hb < 8 g/dl with accompanying hypertension, tachycardia or dizziness	NR	NR	NR	NR	Primary: (1) rate of transfusion; (2) pre- to 24 h post-operative Hb difference; (3) post-operative haematoma requiring revision surgery Secondary: (1) 1-year mortality; (2) post-operative complications (i.e., DVT, cardiac infarction, stroke, pneumonia, urinary tract infection, acute renal failure, deep tissue infection; (3) mean TTS	Primary: (1) Increased need for blood transfusion for DOACs (38.5%) compared to no anticoagulation (16.4%), ASS (21.6%), PAI (26.7%) or VKA (24.0%) (2) No difference for ASS, PAI, DOAC, no anticoagulation for Hb-difference. Patients on VKA had lower post-operative Hb difference (3) No difference for PAI, DOAC, ASS, VKA, no anticoagulation for post-operative haematoma Secondary: (1) No difference for PAI, DOAC, ASS, VKA, no anticoagulation for 1-year mortality (2) No post-operative complications identified (3) No difference for PAI (8.5 h), DOAC (9.5 h), ASS (7.4 h), VKA (10.0 h), no anticoagulation (8.2 h) for TTS
Shani et al. 2021 [53]	NR	Time from arrival in hospital emergency room until surgery	NR	NR	NR	Primary: (1) TTS < 48 h Secondary: (1) TTS; (2) LOS; (3) unadjusted and adjusted 30-day and 6-month mortality	Primary: (1) Lower proportion of DOACs (69.7%) had TTS < 48 h (warfarin: 69.8%) compared to patients not on anticoagulants (89.3%) Secondary: (1) Unadjusted TTS longer for DOACs (1.9 ± 1.6 days) and warfarin (2.0 ± 2.2 days) compared to no anticoagulants (1.3 ± 1.8 days). TSS remained longer for DOACs after adjusting for age, gender and Charlson score (2) LOS longer for DOACs (9.9 ± 9.0 days) and warfarin (9.5 ± 9.3 days) compared to no anticoagulants (8.8 ± 8.6 days) (3) Unadjusted 30-day mortality higher for DOACs (6.0%) and warfarin (10.0%) compared to no anticoagulants (4.2%). Unadjusted 6-month mortality higher for DOACs (16.9%) and warfarin (25.1%) compared to no anticoagulants (13.1%) After adjusting for age, gender and Charlson score, 30-day and 6-month mortality were similar for DOACs vs. no anticoagulants and higher for warfarin compared to DOACs or no anticoagulants
Tarrant et al. 2020 [39]	Peri-operative Hb and admission estimate glomerular filtration rate (eGFR)	TTS from admission and TTS after last DOAC dose (Ts)	General (*n* = 96 DOACs and *n* = 55 controls) Neuraxial (*n* = 15 DOACs and *n* = 56 controls)	NR	NR	Primary: (1) 30-day mortality Secondary: (1) Number of packed cells transfused; (2) Post-operative day (POD) 1 Hb; (3) Serious adverse events (SAE) including myocardial infarction, acute renal failure, respiratory failure, cerebrovascular accident, DVT, PE, pneumonia, bacteraemia/sepsis, surgical site infection, post-operative haemorrhage; (4) TTS from admission; (5) Median LOS (Q1-Q3); (6) inpatient mortality	Primary: (1) No difference in 30-day mortality between DOACs (14%) and controls (6.3%) Secondary: (1) Transfusion not reported by DOACs vs. controls (2) POD1 Hb not reported by DOACs vs. controls (3) SAEs not reported by DOACs vs. controls (4) DOACs higher TTS (1.8 ± 1.3 days) vs. controls (1.2 ± 0.7 days) (5) Longer LOS for DOACs [11 (6.5–18) days] vs. controls [6.9 (4.2–11) days] (6) Higher inpatient mortality for DOAC (7.1%) vs. controls (0.9%)
Tran et al. 2015 [54]	Estimated intra-operative blood loss from anaesthesia record. Blood transfusions Bleeding events as defined by ISTH criteria	Time from admission to surgery	NR	NR	NR	Primary: (1) TTS Secondary: (1) Intra-operative blood loss; (2) blood transfusion; (3) bleeding events; (4) DVT or PE; (5) stroke; (6) in-hospital mortality	Primary: (1) Median TTS longer for DOAC and VKA vs. controls (40 h vs. 26.2 h). Longer TTS for DOAC vs. VKA (66.9 h vs. 39 h) Secondary: (1, 2, 3) No difference in major bleeding episodes for DOAC and VKA vs. controls (4) No difference in DVT or PE for DOAC and VKA vs. controls (5) No difference in stroke for DOAC and VKA vs. controls (6) No difference in in-hospital mortality for DOAC and VKA vs. controls
Viktil et al. 2019 [55]	Difference between last Hb measurement before surgery and the first after surgery	Time from admission to surgery	NR	NR	NR	Primary: (1) serum concentrations; (2) elimination half-life Secondary: (1) TTS; (2) difference in Hb	Primary: (1) 50% of DOAC users had serum concentrations above reference ranges at admission (2) DOACs prolonged elimination half-life (possibly due to reduced renal function and low drug clearance) Secondary: (1) TTS longer for DOAC vs. Warfarin vs. PAI (median 44 vs. 25 vs. 22 h) (2) Hb lower for Warfarin vs. DOACs vs. PAIs. (median 1.1 vs. 2.2 vs. 1.9)

*NR* not reported

The most common health outcomes examined were time to surgery (100%), mortality (95.2%; *n* = 20), blood loss (76.2%; *n* = 16), post-operative complications (52.4%; *n* = 11) and hospital LOS (47.6%; *n* = 10). Type of anaesthesia used during surgery was only recorded in five (23.8%) studies. Information regarding when DOAC use ceased prior to surgery was not often recorded (9.5%; *n* = 2).

### Time to surgery

Time to surgery (either exact or within < 48 h was not defined in six (30.0%) studies. Of the 18 studies that examined exact time to surgery, 12 (57.1%) identified that DOAC users had a longer time to surgery, 5 (23.8%) found no difference in time to surgery, and 1 (4.8%) study identified a longer time to surgery for closed reduction internal fixation, but not for hemiarthroplasty compared to patients not using anticoagulants prior to surgery. Five (83.3%) of six studies identified that DOAC users had a lower proportion of surgeries conducted within 48 h compared to patients not using anticoagulants.

### Blood loss and transfusions

Blood loss definitions varied and none of the nine studies that reported blood loss found a difference in blood loss between patients using DOACs versus no anticoagulants. Only 2 (18.2%) of the 11 studies that reported on the proportion of transfusions between DOACs compared to patients not using anticoagulants found a higher proportion of blood transfusions for patients using DOACs.

### Post-operative complications and hospital LOS

No difference was found in post-operative complication rates for the eight studies that reported on complications and no difference was found for the three studies that reported on the incidence of stroke between DOAC users and patients not using anticoagulants. One (50.0%) of two studies that reported on wound ooze (defined as clinically identified ooze with or without bleeding) found a higher proportion of ooze for DOAC users versus patients not using anticoagulants. One (14.3%) of the seven studies that reported on re-operations/readmissions identified a higher proportion of readmissions for DOAC users, compared to patients not using anticoagulants. Four (40.0%) of the ten studies that reported on hospital LOS identified a higher LOS for DOAC users.

### Mortality

Seven (87.5%) of 8 studies that reported in-hospital mortality, 12 (92.3%) of 13 studies that reported 30-day mortality, and 6 (85.7%) of 7 studies that reported mortality at 1-year identified no difference in mortality rates for DOAC users compared to patients not using anticoagulants. One (14.3%) study identified a higher 1-year mortality for DOAC users for closed reduction internal fixation, but not for hemiarthroplasty compared to patients not using anticoagulants.

### Quality assessment

Methodological quality assessment measures for articles varied and few studies (38.1%; *n* = 8) received all ‘Yes’ ratings (Tables 2 and 3).

**Table 2:** Quality assessment using the CASP Appraisal Checklist for cohort studies

Authors and publication year	Q1	Q2	Q3	Q4	Q5a	Q5b	Q6a	Q6b	Q8	Q9	Q10	Q11	Q12
Bruckbauer et al. 2019 [48]	Y	Y	Y	Y	C	C	NA	NA	Y	Y	Y	Y	Y
Cafaro et al. 2019 [49]	Y	Y	Y	Y	C	C	NA	NA	Y	Y	Y	Y	Y
Creeper et al. 2020 [50]	Y	Y	Y	Y	C	N	Y	Y	Y	Y	Y	Y	Y
Daugaard et al. 2019 [47]	Y	Y	Y	Y	Y	Y	Y	Y	Y	Y	Y	Y	Y
Frenkel Rutenberg et al. 2018 [51]	Y	Y	Y	Y	C	C	Y	Y	Y	Y	Y	Y	Y
Hourston et al. 2020 [42]	Y	Y	Y	Y	Y	Y	Y	Y	Y	Y	Y	Y	Y
King et al. 2020 [38]	Y	Y	Y	Y	Y	Y	Y	Y	Y	Y	Y	Y	Y
Leer-Salvesen et al. 2020 [43]	Y	Y	Y	Y	N	N	Y	Y	Y	Y	Y	Y	Y
Lott et al. 2019 [12]	Y	Y	Y	Y	N	N	NA	NA	Y	Y	Y	Y	Y
Mahmood et al. 2021 [40]	Y	Y	Y	Y	Y	Y	Y	Y	Y	Y	Y	Y	Y
Saliba et al. 2020 [44]	Y	Y	Y	Y	Y	Y	Y	Y	Y	Y	Y	Y	Y
Scherman et al. 2019 [45]	Y	Y	Y	Y	Y	Y	Y	Y	Y	Y	Y	Y	Y
Schuetze et a. 2019 [46]	Y	Y	Y	Y	Y	Y	Y	Y	Y	Y	Y	Y	Y
Shani et al. 2021 [53]	Y	Y	Y	Y	C	C	Y	Y	Y	Y	Y	Y	Y
Viktil et al. 2019 [55]	Y	Y	Y	Y	C	C	NA	NA	N	C	Y	Y	Y

Y—yes; N—no; C—cannot tell; NA— not applicable; CI—confidence intervals

The question “What were the results of this study?” has been presented in Table 1 and is therefore not included in this Table 2

**Table:** CASP Appraisal Checklist questions

1. Did the study address a clearly focussed issue?	6b. Was the follow-up of subjects long enough?
2. Was the cohort recruited in an acceptable way?	7. What are the results of this study?
3. Was the exposure accurately measured to minimise bias?	8. How precise are the results?
4. Was the outcome accurately measured to minimise bias?	9. Do you believe the results?
5a. Have the authors identified all important confounding factors?	10. Can the results be applied to the local population?
5b. Have they taken into account the confounding factors in the design and/or analysis?	11. Do the results of this study fit with other available evidence?
6a. Was the follow-up of the subjects complete enough?	12. Does the study have implications for practice?

**Table 3:** Quality assessment using the CASP Appraisal Checklist for case–control and case-comparison matched studies

Authors and publication year	Q1	Q2	Q3	Q4	Q5	Q6a	Q6b	Q7	Q8	Q9	Q10	Q11
Franklin et al. 2018 [37]	Y	Y	Y	C	Y	Y	Y	C	Y	Y	Y	Y
Gosch et al. 2020 [52]	Y	Y	Y	C	Y	Y	C	C	C	Y	Y	Y
Mullins et al. 2018 [26]	Y	Y	Y	C	Y	Y	Y	C	Y	Y	Y	Y
Rostagno et al. 2021 [41]	Y	Y	Y	Y	Y	Y	C	C	C	Y	Y	Y
Tarrant et al. 2020 [39]	Y	Y	Y	Y	Y	Y	Y	C	Y	Y	Y	Y
Tran et al. 2015 [54]	Y	Y	Y	Y	Y	Y	C	C	C	Y	Y	Y

Y—yes; N—no; C—cannot tell; NA—not applicable; CI—confidence intervals

**Table:** CASP Appraisal Checklist questions

1. Did the study address a clearly focussed issue?	6b. Have the authors taken account of the potential confounding factors in the design and/or in their analysis?
2. Did the authors use an appropriate method to answer their question?	7. How large was the treatment effect?
3. Were the cases recruited in an acceptable way?	8. How precise was the estimate of the treatment effect?
4. Were the controls selected in an acceptable way?	9. Do you believe the results?
5. Was the exposure accurately measured to minimise bias?	10. Can the results be applied to the local population?
6a. Aside from the experimental intervention, were the groups treated equally?	11. Do the results of this study fit with other available evidence?

## Discussion

This rapid review identified 21 studies that examined the impact of pre-operative DOAC use on hip fracture patients’ health outcomes. Overall, in these studies the effect of DOAC use on hip fracture patient health outcomes compared to patients not taking any anticoagulants was mixed. Compared to patients not taking anticoagulants, in 57.1% of studies DOAC users had a longer time to surgery, 40.0% had a longer hospital LOS, 18.2% had a higher proportion of blood transfusions, and 14.3% had a higher proportion of readmissions, where each of these outcomes was examined. No difference was identified in overall blood loss, post-operative complication rates, stroke, or in-hospital, 30-day or 1-year mortality between DOAC users and patients not taking any anticoagulants in studies that examined these patient outcomes.

The advantages of using DOACs are that they are taken orally and are associated with fewer dietary and other medication interactions [10, 23]. DOACs have a fast therapeutic onset, lower complication and monitoring requirements, and are more cost-effective compared to VKAs, such as warfarin [10, 24]. A systematic review and meta-analysis that examined the effect of DOAC or VKA use on time to surgery and mortality for hip fracture patients found that time to surgery was 15.5 h longer for patients taking DOACs than patients not on anticoagulants, with no difference in time to surgery for patients taking DOACs versus VKAs, and that there was no difference in in-hospital mortality for patients taking DOACs compared to patients not on anticoagulants [25].

While this rapid review found that time to surgery was reported as longer for patients using DOACs pre-operatively, whether or not the surgery was conducted within 48 h was only examined by one-third of studies, with five of six studies identifying a lower proportion of surgery conducted within 48 h for patients taking DOACs versus no anticoagulants. Prior research has largely indicated that better health outcomes after hip fracture surgery are associated with surgery that is conducted within 48 h of patient admission [5–8]. In one study, hip fracture surgery for DOAC users within 24 h of admission was not associated with increased blood loss, transfusion rates or 30-day mortality compared to matched patients not taking anticoagulants [26]. However, larger population-based studies are needed to further examine patient outcomes for DOAC users undergoing surgery < 24 h after admission.

The current review found no difference in overall blood loss or post-operative complication rates, and only two studies consistently identified higher mortality rates for hip fracture patients taking DOACs versus no anticoagulants. Prior systematic reviews and meta-analyses of health outcomes of DOACs users compared to patients taking VKAs not limited to trauma found that DOAC users had a reduced patient complication and mortality risk, and showed no difference in blood loss [27–30], except for dabigatran which was associated with a higher risk of gastrointestinal bleeding compared to VKAs [31]. That there was no difference found for patients taking DOACs versus no anticoagulants for mortality, post-operative complications or blood loss may stem in part from a delayed time to surgery for patients taking DOACs as surgical teams aim to optimise these patients prior to surgery to achieve the best outcomes possible. Given a randomised controlled trial is unlikely to take place, and there are large clinical hip fracture registries in a number of countries which measure high-level outcomes in hip fracture patients, developing a consensus and monitoring approach for time to surgery for patients taking DOACs may guide practice in the future.

Published reviews and practical guidelines have indicated there is no consensus on an appropriate DOAC free period prior to acute hip fracture surgery [32–34]. The recommended time to surgery in guidelines has ranged from 12 h after the last dose to up to 4 days, depending on half-life of the DOAC and patient renal function [32–35]. Most recommendations on time to surgery for patients pre-operatively taking DOACs have been made for patients undergoing elective surgery [32], and there has been limited examination of DOACs and time to surgery in the acute care setting, such as for hip fracture.

This systematic rapid review has identified that the research evidence surrounding pre-operative DOAC use and time to surgery is mainly derived from retrospective cohort studies conducted at single facilities which describe current varied practice. The review identified limited evidence to support the development of practical clinical guidelines on the management of hip fracture patients taking DOACs. These studies indicate that almost two-thirds of DOAC users are delayed to hip fracture surgery compared to patients not taking any anticoagulants. Whether a delay to surgery for hip fracture patients taking DOACs pre-operatively is justified needs further clarification from robust, large population-based studies [32, 35], along with further pragmatic investigation of the use of reversal agents for DOACs prior to acute hip fracture surgery [36].

This rapid review has also shown there is a need for consistency in the type of health outcomes examined post-hip fracture surgery to determine the effect of DOACs on patient outcomes. This includes providing clear definitions for each health outcome examined, particularly for the measurement of blood loss, noting the hours prior to surgery that DOAC use ceased, the type of anaesthesia used during surgery, and the specific type of post-operative complications examined. In six (33.3%) studies in this review [26, 37–41], a matched comparison group was used or data were matched post hoc, but matching criteria were not consistent across studies, nor were consistent comparator groups used. Where matching was not used, only six (40.0%) studies specified and adjusted for potential covariates [42–47], indicating potential limitations of sample size to conduct regression analyses. In ten (47.6%) studies, there were less than 50 patients taking DOACs.

With the ageing population and the growth of chronic diseases, the use of DOACs in older hip fracture patients is likely to increase [9]. Further pragmatic research is needed to examine the impact of pre-operative DOAC use on hip fracture patient health outcomes, including examining patient experience measures along with patient-reported outcome measures, particularly regarding HRQoL and ADLs.

The strengths of this rapid review were that it followed the PRISMA guidelines, it used a comprehensive keyword search strategy involving three databases, a university medical librarian assisted with the development of the keyword search terms, and multiple reviewers were involved in the data extraction phase with high interrater reliability. Any clarifications or disagreements were discussed between reviewers and consensus was obtained. However, there were some limitations of the review. Studies published in non-English languages were excluded, which may result in language bias. The rapid review did not examine clinical trials registries, so any trials currently underway were excluded. Three studies included patients aged < 65 years; however the mean patient age in these studies was in the 80 s, warranting their inclusion.

## Conclusions

This review found limited evidence to support guidelines on the management of hip fracture patients taking DOACs. The effect of DOAC use on hip fracture patient health outcomes compared to patients not taking any anticoagulants was mixed, although patients on DOACs had a longer time to surgery. It highlights the need for robust, population-based studies and the consistent examination of hip fracture surgery health outcomes to determine the most appropriate management of patients with a hip fracture taking a DOAC.

### Electronic supplementary material

Below is the link to the electronic supplementary material.

Supplementary file1 (DOCX 38 KB)

## Data Availability

All data generated or analysed during this study are included in this published article.

## Appendix 1: Search strategy for each database

### Medline

“Exp Anticoagulants/” OR “Administration, Oral/” OR “Hip Fractures/su [Surgery]” OR “1 and 3” AND “limit 4 to "all aged (65 and over)" AND limit 5 to (“middle aged (45 plus years)” OR “all aged (65 and over)”) AND “limit 6 to (English language and (clinical trial, all” OR “randomized controlled trial” OR “systematic review”)).

### Embase

“Exp hip fracture” OR “exp anticoagulant agent/” OR “1 and 2” OR “exp postoperative period/” OR “3 and 4” OR “(surgery or surgical).tw.” OR “1 and 6” OR “2 and 7” OR “4 and 8” AND “limit 9 to (English language and (clinical trial or randomized controlled trial))” AND “limit 12 to (English language and yr = “2011–2022”)”.

### Scopus

(TITLE-ABS-KEY (anticoagulant* OR “oral anticoagulant” ORr “DOAC*” OR Rivaroxaban OR Apixaban OR Dabigatran OR Edoxaban) AND ABS (“hip fracture” OR “hip surgery”) AND ABS (postoperative* OR mortality OR “time to surgery” OR “length of stay” OR “complication*” OR bleed* OR “blood loss” OR embolism OR thrombosis OR “intensive care*”)) AND (LIMIT-TO (LANGUAGE, “English”)).
