# Supplementary material for: Synthesis of the evidence on the impact of pre-operative direct oral anticoagulants on patient health outcomes after hip fracture surgery: rapid systematic review
Source: Eur J Trauma Emerg Surg. 2022 Mar 11;48(4):2567–87. doi: 10.1007/s00068-022-01937-8 (PMC9360144; doi:10.1007/s00068-022-01937-8)
Supplement: Supplementary file 1 — Supplementary file1 (DOCX 38 KB) [file 68_2022_1937_MOESM1_ESM.docx]

**Supplementary** **Table 1: Narrative synthesis of the most common health outcomes examined for DOAC users versus no anticoagulant use prior to hip fracture surgery**

|  | **Time to surgery (TTS)** | | **Blood loss** | | **Complications** | | | **LOS** | **Re-operation/**  **Readmission** | | **Mortality** | | |
| --- | --- | --- | --- | --- | --- | --- | --- | --- | --- | --- | --- | --- | --- |
| **Authors**^1^ | **Longer TTS** | **Lower %<48h** | **Higher blood loss** | **Higher % transfusions** | **Higher complicat-ions** | **Higher wound ooze** | **Higher stroke** | **Higher LOS** | **Higher re-operation** | **Higher readmiss-ions** | **Higher in-hospital** | **Higher 30-day** | **Higher 1-year** |
| Bruckbauer et al [48] | Y | - | N | - | - | - | - | - | - | - | N | - | - |
| Cafaro et al [49] | Y | Y | - | N | - | - | N | N | - | - | N | - | - |
| Creeper et al 2020 [50] | Y | - | N | N | - | - | - | - | - | - | - | N | - |
| Daugaard et al 2019 [47] | - | - | - | Y | - | - | - | - | - | - | - | N | - |
| Franklin et al [37] | Y | - | N | N | N | - | - | N | - | Y | N | N | N |
| Frenkel Rutenberg et al [51] | - | Y | - | N | N | - | - | - | - | N | N | - | N |
| Gosch et al 2020 [52] | Y | - | N | N | N | - | N | Y | N | - | - | N | - |
| Hourston et al [42] | Y | - | - | - | - | - | - | N | - | - | - | N | N |
| King et al [38] | Y | - | N | N | N | - | - | N | N | - | - | N | - |
| Leer Salvesen et al [43] | N | - | N | N | - | Y | - | N | - | N | N | N | - |
| Lott et al [12] | N | - | N | N | N | - | - | - | - | - | - | - | - |
| Mahmood et al [40] | N | - | - | Y | N | N | - | - | N | - | - | Y | Y |
| Mullins et al [26] | N | - | - | N | - | - | - | - | N | - | - | N | - |
| Rostagno et al [41] | Y | Y | - | N | N | - | - | N | - | - | N | - | - |
| Saliba et al [44] | - | Y | N | N | - | - | - | Y | - | - | - | N | N |
| Scherman et al [45] | P | N | - | N | - | - | - | - | - | - | - | N | P |
| Schuetze et al [46] | N | - | - | Y | N | - | - | - | - | - | - | - | N |
| Shani et al [53] | Y | Y | - | - | - | - | - | Y | - | - | - | N | - |
| Tarrant et al [39] | Y | - | - | - | - | - | - | Y | - | - | Y | N | - |
| Tran et al [54] | Y | - | N | - | N | - | N | - | - | - | N | - | - |
| Viktil et al [55] | Y | - | - | - | - | - | - | - | - | - | - | - | - |

Y=Yes; N=No; P=partial, longer TTS and higher 1-year mortality for closed reduction internal fixation, but not hemiarthroplasty.

^1^Most common DOAC health outcomes compared to no-anticoagulant use, except Creeper et al where DOAC users were compared to Warfarin, Franklin et al where control group criteria was unspecified, Lott et al where DOAC users were compared to PAI, and Viktil et al where DOAC users were compared to Warfarin users.
